# Cerebral vascular amyloid seeds drive amyloid β-protein fibril assembly with a distinct anti-parallel structure

**DOI:** 10.1038/ncomms13527

**Published:** 2016-11-21

**Authors:** Feng Xu, Ziao Fu, Sharmila Dass, AnnMarie E. Kotarba, Judianne Davis, Steven O. Smith, William E. Van Nostrand

**Affiliations:** 1 Departments of Neurosurgery and Medicine, Stony Brook University, Stony Brook, New York 11794, USA; 2 Department of Biochemistry and Cell Biology, Stony Brook University, Stony Brook, New York 11794, USA

## Abstract

Cerebrovascular accumulation of amyloid β-protein (Aβ), a condition known as cerebral amyloid angiopathy (CAA), is a common pathological feature of patients with Alzheimer's disease. Familial Aβ mutations, such as Dutch-E22Q and Iowa-D23N, can cause severe cerebrovascular accumulation of amyloid that serves as a potent driver of vascular cognitive impairment and dementia. The distinctive features of vascular amyloid that underlie its unique pathological properties remain unknown. Here, we use transgenic mouse models producing CAA mutants (Tg-SwDI) or overproducing human wild-type Aβ (Tg2576) to demonstrate that CAA-mutant vascular amyloid influences wild-type Aβ deposition in brain. We also show isolated microvascular amyloid seeds from Tg-SwDI mice drive assembly of human wild-type Aβ into distinct anti-parallel β-sheet fibrils. These findings indicate that cerebrovascular amyloid can serve as an effective scaffold to promote rapid assembly and strong deposition of Aβ into a unique structure that likely contributes to its distinctive pathology.

## Abstract (web summary)

Cerebrovascular accumulation of Aβ is a common feature of Alzheimer's disease, though it is unclear whether mutant vascular amyloid is capable of Aβ seeding. Here, the authors show microvascular amyloid seeds are capable of driving wild-type Aβ to assemble into distinctive anti-parallel fibrillary structures.

## Graphical abstract

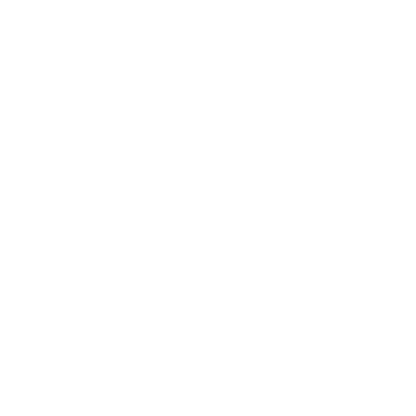

Cerebral amyloid angiopathy (CAA) is a prominent cerebral vascular condition that can cause vascular cognitive impairment and dementia (VCID)^1^,^2^. The most common form of CAA results from the accumulation of the amyloid β-protein (Aβ) and is present at varying levels in >80% of elderly individuals^1^,^2^,^3^,^4^. Although the Aβ peptide is most recognized for its accumulation in parenchymal amyloid plaques, its parallel deposition in the cerebral vasculature is commonly observed in the brains of patients with Alzheimer's disease (AD)^3^,^5^,^6^. Besides AD and spontaneous forms of CAA, several familial types of CAA have been identified that are caused from specific mutations that occur within the Aβ peptide. For example, the Dutch-type of familial CAA is caused by an E22Q mutation in Aβ and results in early development of extensive cerebral vascular amyloid deposition that promotes cerebral haemorrhages at mid-life and VCID^7^,^8^,^9^. Likewise, the Iowa-type of familial CAA is caused by a D23N mutation in Aβ that similarly causes early-onset deposition of cerebral vascular amyloid with patients also exhibiting VCID^10^,^11^. Considering the pathology of familial CAA, it is noteworthy that patients generally harbor only one mutant *APP* allele while the other allele is normal resulting in the production of both CAA mutant and wild-type Aβ peptides in the brain. How CAA mutant amyloid influences the assembly and structure of wild-type Aβ peptides to exclusively form vascular fibrillar amyloid is poorly understood.

Cerebral vascular amyloid can contribute to cognitive decline in AD and familial forms of CAA by promoting several pathological consequences including neuroinflammation^12^,^13^, chronic hypoperfusion and ischemia^14^,^15^, and, in severe cases, loss of vessel wall integrity and haemorrhage^16^,^17^. Further, studies have reported that cerebral microvascular Aβ deposition is more often correlated with dementia in individuals afflicted with AD and spontaneous CAA disorders^4^,^18^. Indeed, recent findings in experimental mouse models suggest that even small amounts of cerebral microvascular amyloid deposition, that precedes high parenchymal levels of Aβ, are sufficient to drive VCID underscoring the importance of CAA^19^. Yet despite these deleterious consequences that are unique to vascular amyloid there is a fundamental gap in understanding what causes the unique pathology in CAA.

Regardless of whether it is wild-type Aβ found in sporadic CAA/AD or mutant forms of Aβ found in familial CAA, cerebrovascular amyloid appears to exhibit some distinct differences compared with parenchymal plaque amyloid. For example, parenchymal plaques are largely composed of Aβ42 whereas cerebral vascular amyloid is mainly the shorter Aβ40 species^3^,^4^. Also, antibodies were identified that appear to selectively recognize vascular amyloid and not parenchymal amyloid^20^. Finally, a recent study reported that analogues of the dye resorufin preferentially bound to vascular amyloid deposits in brain^21^. Together, these studies suggest that structural differences might exist between cerebrovascular amyloid and parenchymal plaque amyloid and that perhaps these differences account for the specific developmental aspects of CAA and some of the unique pathological responses to CAA that result in VCID.

Here, we first show *in vitro* that CAA mutant Aβ can adopt parallel or anti-parallel β-sheet structures and that fibril seeds from both can promote rapid fibril assembly of wild-type Aβ peptides with the corresponding fibrillar signatures. Two approaches are then taken to demonstrate that CAA mutant vascular amyloid similarly influences wild-type Aβ deposition in the brains of transgenic mice. First, intra-hippocampal administration of biotin-labelled wild-type Aβ peptides results in strong accumulation on pre-existing cerebral microvascular amyloid deposits in Tg-SwDI mice, a model of early-onset CAA mutant microvascular amyloid^22^,^23^. Second, Tg-SwDI mice bred with Tg2576 mice, a model that produces high amounts of human wild-type Aβ in brain^24^, exhibit markedly elevated accumulation of microvascular fibrillar amyloid compared to either single transgenic line. The volume of microvascular CAA in the Tg-SwDI/Tg2576 bigenic mice is dramatically increased and largely composed of human wild-type Aβ. Notably, microvascular amyloid seeds isolated from Tg-SwDI mice can direct the assembly of human wild-type Aβ into an alternative anti-parallel fibril configuration. These findings suggest that early-onset and focal CAA mutant microvascular amyloid can act as a scaffold to promote the robust fibrillar assembly and accumulation of wild-type Aβ with a distinct anti-parallel signature.

## Results

### Solution structure of CAA mutant amyloid fibrils

The predominant fibril form of Aβ observed in solution^25^ and obtained through seeding experiments of brain amyloid^26^ contains cross β-sheet structure in which the individual β-strands have a parallel, in-register orientation. In contrast, previous studies showed that the CAA mutant Aβ40-Iowa (Aβ40-I) sequence can form fibrils with both parallel and anti-parallel structure^27^,^28^. It was found that the anti-parallel form of Aβ40-I was thermodynamically metastable with respect to the parallel form. Here, we first show that the Dutch/Iowa CAA mutant Aβ40 peptide (Aβ40-DI) can also form both parallel and anti-parallel structures in solution. Including both the Dutch-type and Iowa-type mutations together in the same Aβ peptide enhances its fibrillogenic and pathogenic properties *in vitro*^29^.

**Figure 1:**
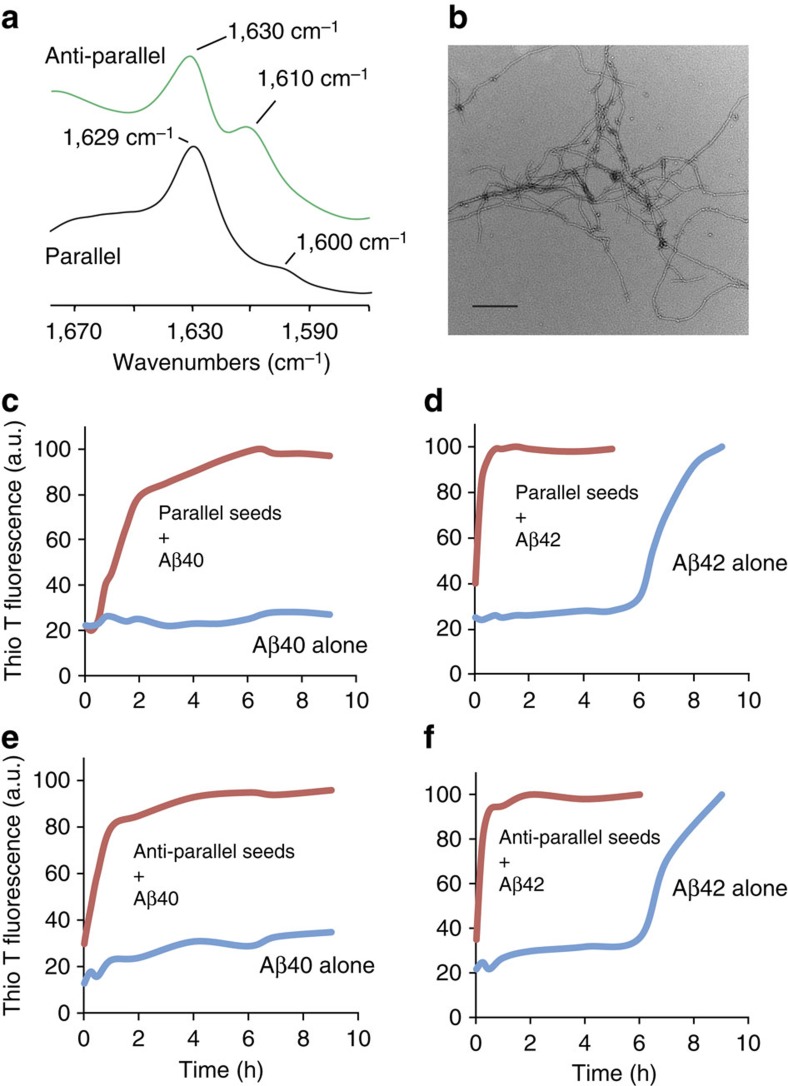
Aβ40-DI CAA mutant amyloid fibril seeds promote the rapid assembly of wild-type Aβ40 and Aβ42 fibrils. (**a**) FTIR spectra of Aβ40-DI having anti-parallel (green) and parallel (black) β-sheet secondary structure. The anti-parallel fibrils were formed at 4-6 °C using the protocol of Tycko^22^,^23^ (Supplementary Fig. 2) and are stable to at least 22 °C (Supplementary Fig. 3). (**b**) TEM of anti-parallel Aβ40-DI obtained at 22 °C. Scale bar, 150 nm. Freshly prepared solutions of wild-type Aβ40 (**c**,**d**) or wild-type Aβ42 (**e**,**f**) were incubated in the absence (blue) or presence (red) of parallel (**c**,**e**) or anti-parallel (**d**,**f**) Aβ40-DI fibrillar seeds. The rate and extent of fibril formation was assessed by thioflavin T fluorescence measurements at 37 °C. TEM, transmission electron microscopy.

In our studies, parallel and anti-parallel β-sheet structures are distinguished by Fourier transform infrared (FTIR) spectroscopy on the basis of isotope-induced shifts and intensity changes observed in the amide I vibration upon specific ^13^C labelling (Supplementary Fig. 1)^30^,^31^. The amide I region of the FTIR spectrum is shown in Fig. 1a for Aβ40-DI fibrils having anti-parallel (green) and parallel (black) β-sheet structure. The different fibril forms were obtained using the protocols developed by Tycko^27^,^28^ and colleagues (Supplementary Fig. 2). The ∼1,630 cm^−1^ band observed in these spectra is characteristic of β-sheet secondary structure. In addition, the Aβ peptide used in these experiments contains a specific ^13^C=O label at Gly33, a residue within the hydrophobic C-terminus that typically forms β-sheet in Aβ fibrils. The ^13^C substitution lowers the frequency of the C=O stretching coordinate and results in isotope-shifted resonances at 1,610 and 1,600 cm^−1^, which differ in intensity depending on whether the β-strands hydrogen-bond in a parallel or anti-parallel orientation. The increase of intensity at 1,610 cm^−1^ relative to 1,600 cm^−1^ is a signature of anti-parallel β-sheet^30^.

The anti-parallel fibrils of Aβ40-DI prepared *in vitro* are metastable and have highly branched, curved structures (Fig. 1b) as previously observed for anti-parallel Aβ40-I fibrils^27^,^28^. They convert to parallel fibrils upon heating to 37 °C (Supplementary Fig. 3). This transition is distinct from the appearance of a transient non-fibrillar intermediate containing anti-parallel β-hairpin structure at 37 °C (Supplementary Fig. 4), which appears before the strong increase of thioflavin T fluorescence characteristic of fibril formation.

We next used parallel and anti-parallel fibrillar seeds of Aβ40-DI to determine their influence on wild-type Aβ40 and Aβ42 fibrillar assembly. As shown in Fig. 1, over the course of 9 h there was little, if any, fibril assembly of wild-type Aβ40 as measured by increased thioflavin T fluorescence. In contrast, either parallel (Fig. 1c) or anti-parallel (Fig. 1d) Aβ40-DI fibril seeds promoted the rapid assembly of wild-type Aβ40 within ≈4 h. Similarly, the more fibrillogenic wild-type Aβ42 promptly assembled within 30 min in the presence of either parallel or anti-parallel Aβ40-DI fibril seeds compared to >8 h in the absence of seeds (Fig. 1e,f, respectively). These findings indicate that both parallel and anti-parallel CAA mutant amyloid seeds can act as a scaffold to rapidly bind wild-type Aβ peptides and strongly promote their assembly into fibrils.

**Figure 2:**
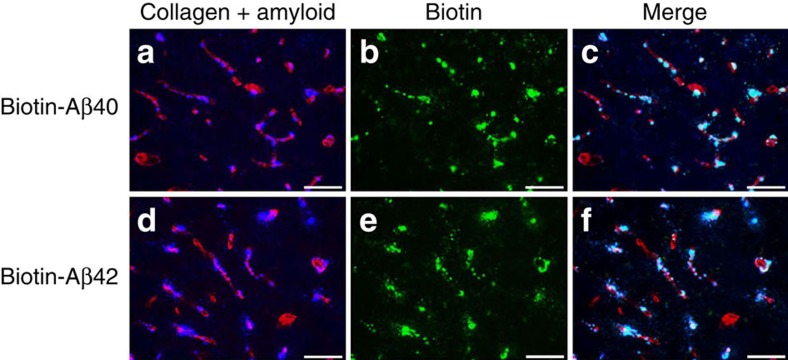
Dutch/Iowa CAA mutant cerebral microvascular amyloid in Tg-SwDI mice promotes the accumulation of administered biotin-labelled wild-type Aβ peptides. Biotin-labelled wild-type Aβ40 (**a**–**c**) or biotin-labelled wild-type Aβ42 (**d**–**f**) were injected into the hippocampal region of 12-month-old Tg-SwDI mice. Brain sections were prepared and fibrillar amyloid was detected using Amylo-Glo (blue) and immunolabelled with an antibody to collagen IV for detection of cerebral blood vessels (red). Biotin-labelled wild-type Aβ40 or Aβ42 peptides were detected using streptavidin-Alexa Fluor 488 (green). Scale bars, 50 μm.

We next investigated how wild-type Aβ peptides interact with early-onset cerebral microvascular amyloid deposits comprised of CAA mutant Aβ *in vivo*. Biotin-labelled human wild-type Aβ40 or Aβ42 peptides were injected into the hippocampal region of twelve months old Tg-SwDI mice, a model that exhibits prominent cerebral microvascular amyloid deposition. The injected biotin-labelled human wild-type Aβ40 (Fig. 2a–c) or Aβ42 (Fig. 2d–f) peptides showed strong and preferential accumulation on pre-existing cerebral microvascular fibrillar amyloid deposits in the surrounding tissue. No accumulations were found when biotin alone was infused into similarly aged Tg-SwDI mice or when biotin-labelled wild-type Aβ peptides were infused into similarly aged wild-type mice without microvascular CAA (Supplementary Fig. 5). These results indicate that familial CAA mutant microvascular amyloid can serve as scaffold for rapid and robust co-deposition of wild-type Aβ peptides in brain.

### Cerebral microvascular amyloid in Tg-SwDI/Tg2576 mice

Next, we sought to determine the outcome of producing high amounts of non-mutated, wild-type human Aβ peptides in the presence of early-onset familial CAA mutant cerebral microvascular amyloid in the brains of transgenic mice. Tg-SwDI mice, which express low levels of human AβPP and produce low amounts of human Dutch/Iowa CAA mutant Aβ (refs 22, 23), were bred with Tg2576 mice, which comparatively express very high levels of human AβPP and produce very high amounts of human wild-type Aβ (ref. 24), to generate bigenic and single transgenic animals. The resulting bigenic Tg-SwDI/Tg2576 mice recapitulate the *in vitro* conditions of Fig. 1, where we have small amounts of CAA mutant cerebral microvascular amyloid seeds in the presence of excess wild-type Aβ peptides. After aging 6–18 months, quantitative ELISAs were performed to measure human Aβ levels in forebrain tissue homogenates prepared from each line of mice (Supplementary Fig. 6). All mice showed a progressive accumulation of Aβ40 and Aβ42 peptides in brain. As the mice aged to 18 months, Tg2576 mice accumulated more Aβ40 and Aβ42 than Tg-SwDI mice. Most notably, there was an exaggerated accumulation of Aβ40 in the bigenic Tg-SwDI/Tg2576 mice that surpassed the additive amounts of Aβ40 observed in each single transgenic line.

**Figure 3:**
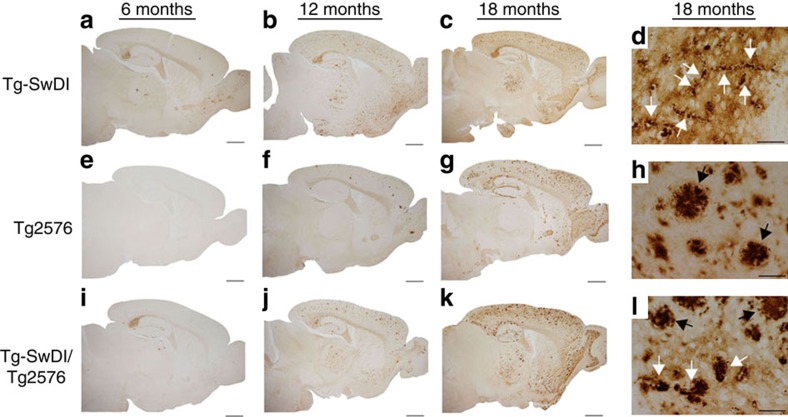
Progressive accumulation of Aβ in transgenic mouse brains. Representative images of 6–18-month-old transgenic mouse brain sections were immunostained for Aβ (brown) as described in Methods. (**a**–**d**) Tg-SwDI mice; (**e**–**h**) Tg2576 mice; and (**i**–**l**) bigenic Tg-SwDI/Tg2576 mice. High-magnification images show vascular deposits (white arrows) and parenchymal plaques (black arrows). Scale bars: low mag, 1 mm; high mag, 50 μm.

The analysis of cerebral human Aβ deposition by immunolabelling showed that at 6 months of age Tg-SwDI mice and bigenic Tg-SwDI/Tg2576 mice exhibited early stage Aβ deposition most prominent in the subiculum region (Fig. 3a,i, respectively), whereas the Tg2576 mice showed little, if any, Aβ deposition at this age (Fig. 3e). However, as all three lines of mice aged from 12 to 18 months the accumulation of deposited Aβ increased dramatically and was most robust in the bigenic Tg-SwDI/Tg2576 mice, reflecting the Aβ ELISA results (Supplementary Fig. 6). Higher magnification images at 18 months showed that Tg-SwDI mice exhibited numerous small, dense deposits along cerebral capillaries and abundant diffuse Aβ deposits in the surrounding brain parenchyma (Fig. 3d), consistent with previous findings^22^,^23^. In contrast, Tg2576 mice showed large, dense parenchymal Aβ plaques (Fig. 3h), while bigenic Tg-SwDI/Tg2576 mice displayed a mixture of parenchymal Aβ plaques and, notably, greatly enlarged Aβ deposits along the capillaries (Fig. 3l).

**Figure 4:**
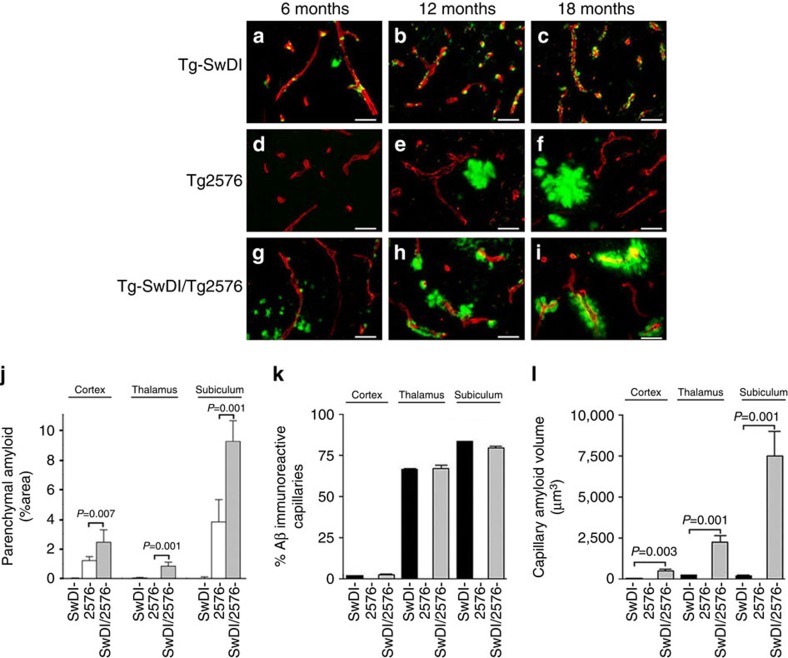
Increased cerebral capillary amyloid deposition in bigenic Tg-SwDI/Tg2576 mouse brain. Transgenic mouse brain sections (from 6 to 18 months old) were stained for fibrillar amyloid using thioflavin S (green) and immunolabelled for cerebral blood vessels using an antibody to collagen IV (red). (**a**–**c**) Tg-SwDI mice; (**d**–**f**) Tg2576 mice; and (**g**–**i**) bigenic Tg-SwDI/Tg2576 mice. Scale bar, 25 μm. Transgenic mouse brain sections (from 18 months old) were stained for fibrillar amyloid using thioflavin S and immunolabelled for cerebral blood vessels using an antibody to collagen IV. (**j**) The area occupied by parenchymal fibrillar amyloid plaques was determined in specific brain regions of each transgenic mouse line using stereological principles as described in Methods. The data presented are the mean±s.d. of 4–6 mice per group. (**k**) The frequency of cerebral capillary amyloid was determined in specific brain regions of each transgenic mouse line using stereological principles as described in Methods. The data presented are the mean±s.d. of 4–6 mice per group. (**l**) Microvessels exhibiting amyloid deposition were selected from distinct brain regions of each transgenic mouse line and the volume of microvascular amyloid was determined as described in Methods. The data presented are the mean±s.d. of a total of 50 microvessels selected from each brain region of three mice per group. Pair-wise comparisons were made using *t*-test and significant differences (*P*<0.05) are indicated and limited to three decimal places.

To specifically identify fibrillar amyloid deposition in the brains of the different mice we performed thioflavin S staining. Examination of the brain tissues revealed striking differences in the compartmental pattern of fibrillar amyloid deposition. Tg-SwDI mice, from 6 months of age on, showed the characteristic pattern of progressive accumulation of fibrillar amyloid in the form of small punctate deposits primarily along cerebral capillaries (Fig. 4a–c). In contrast, Tg2576 mice showed no appreciable fibrillar amyloid deposition at 6 months of age (Fig. 4d). However, as the Tg2576 mice continued to age there was progressive accumulation of primarily large, fibrillar amyloid parenchymal plaques, but no appreciable microvascular amyloid (Fig. 4e,f). On the other hand, the bigenic Tg-SwDI/Tg2576 mice exhibited a mixed pattern of fibrillar amyloid deposition. At the early age of 6 months small capillary and parenchymal amyloid deposits appeared (Fig. 4g). As the bigenic mice continued to age from 12 to 18 months parenchymal amyloid plaques were evident but the striking feature was the notably enlarged accumulation of fibrillar amyloid around the cerebral capillaries (Fig. 4h,i).

The compartmental accumulation of fibrillar amyloid in several brain regions of the different transgenic lines at eighteen months of age was determined using quantitative stereological measures. There was a near doubling in the amount of parenchymal plaque fibrillar amyloid in the bigenic Tg-SwDI/Tg2576 mice compared with Tg2576 mice, whereas Tg-SwDI exhibited little appreciable parenchymal fibrillar amyloid (Fig. 4j). On the other hand, there was very high capillary CAA in the Tg-SwDI mice, particularly in the thalamus and subiculum, but little, if any, capillary CAA in Tg2576 mice (Fig. 4k). Interestingly, in the bigenic Tg-SwDI/Tg2576 mice the number of capillaries affected with CAA was essentially the same as in the Tg-SwDI mice in all brain regions. Subsequently, the volume of cerebral capillary amyloid in each of the transgenic lines was determined by measuring the volume of fibrillar amyloid surrounding the capillaries and then subtracting the volume of the affected capillaries (Fig. 4l). Although the frequency of capillary CAA is very high in Tg-SwDI mice the actual volume of this amyloid is quite low as these deposits are typically small and punctate in this model (see Fig. 4a–c). In contrast, the volume of the capillary amyloid deposits in the bigenic Tg-SwDI/Tg2576 mice was dramatically increased from 10- to 30-fold compared with Tg-SwDI mice.

### Increase of wild-type Aβ in bigenic Tg-SwDI/Tg2576 mice

**Figure 5:**
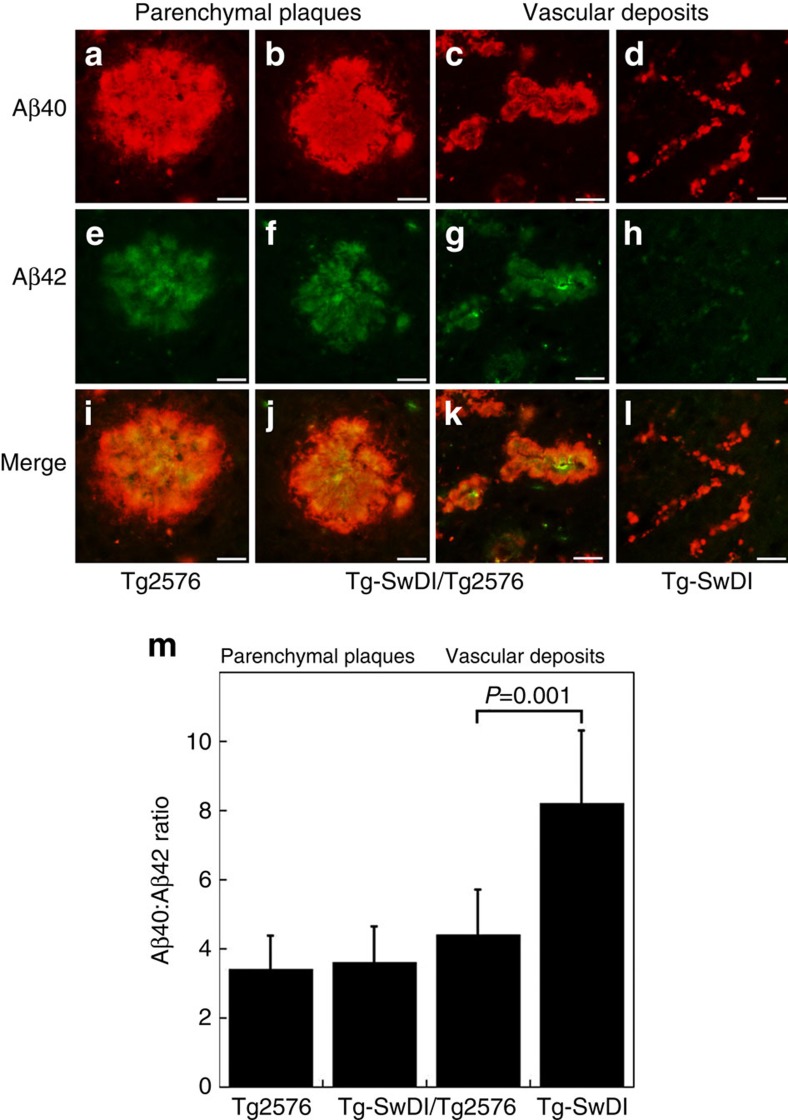
Quantitative immunohistochemical analysis of cerebral Aβ40 and Aβ42 deposition in the different transgenic mice. Brain sections obtained from the different transgenic mouse lines at 18 months of age were double immunolabelled for Aβ40 (red) (**a**–**d**) and Aβ42 (green) (**e**–**h**) and images were merged (**i**–**l**). Scale bars, 20 μm. (**m**) The levels of Aβ40 and Aβ42 in parenchymal plaque and capillary deposits were determined in the different transgenic lines and expressed as the ratio of Aβ40:Aβ42. The data presented are the mean±s.d. of 15 deposits per each line of mice. Pair-wise comparisons were made using *t*-test and significant differences (*P*<0.05) are indicated and limited to three decimal places.

The composition of Aβ in the vascular and parenchymal deposits in each line of mice was evaluated. First, brain sections from each line of mice were immunolabelled with antibodies specific for either Aβ40 or Aβ42 and the relative levels of each Aβ species in each type of deposit was determined. The composition of Aβ40 and Aβ42 in parenchymal plaque deposits in Tg2576 mice or bigenic Tg-SwDI/Tg2576 was essentially the same (Fig. 5). In contrast, the vascular deposits in Tg-SwDI mice contained much higher amounts of Aβ40 (≈90%) consistent with earlier reports^22^,^23^,^32^. In comparison, the enlarged vascular deposits in bigenic Tg-SwDI/Tg2576 mice contained significantly higher amounts of Aβ42 than the vascular deposits in Tg-SwDI mice.

**Figure 6:**
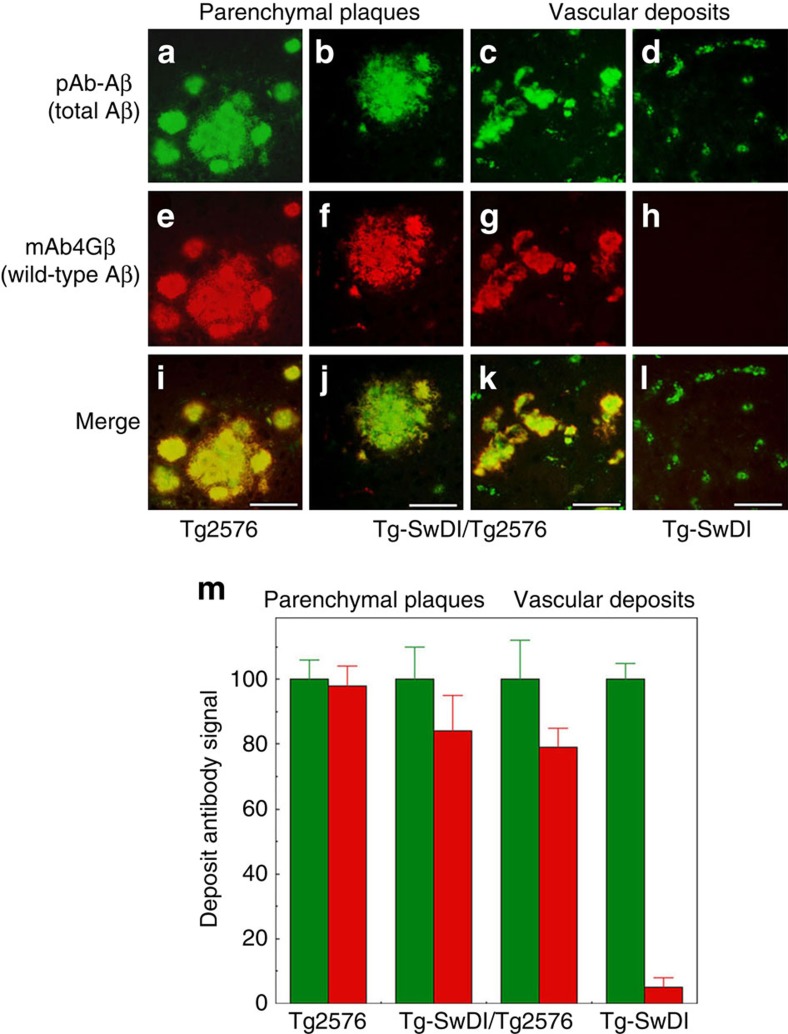
Quantitative immunohistochemical analysis of cerebral human wild-type Aβ deposition in the different transgenic mice. Brain sections obtained from the different transgenic mouse lines at eighteen months of age were double immunolabelled for human Aβ using a polyclonal anti-Aβ (pAb-Aβ) antibody which recognizes both wild-type and CAA mutant Aβ, (green) (**a**–**d**) and mAb4G8, which recognizes wild-type Aβ, but not CAA mutant Aβ, (red) (**e**–**h**) and images were merged (**i**–**l**). Scale bars, 50 μm. (**m**) The levels of pAb-Aβ-positive total Aβ (green) and mAb4G8-positive wild-type Aβ (red) in parenchymal plaque and capillary deposits were determined in the different transgenic lines. The data presented are the mean±s.d. of 15 deposits per each line of mice.

Subsequently, we determined whether CAA mutant or wild-type Aβ accumulation was responsible for the large increase in capillary amyloid volume observed in the bigenic Tg-SwDI/Tg2576 mice. To differentiate between human wild-type Aβ and human Dutch/Iowa CAA mutant Aβ peptides, we performed immunolabelling using the mAb4G8 antibody, which recognizes a mid-region epitope on human wild-type Aβ peptides^33^. The presence of the E22Q Dutch and D23N Iowa mutations abolishes the 4G8 epitope in human Aβ as confirmed by dot blot analysis (Supplementary Fig. 7). On the other hand, the rabbit polyclonal antibody directed towards an N-terminal epitope on Aβ (pAb-Aβ)^34^ equally recognizes wild-type and CAA mutant human Aβ peptides (Supplementary Fig. 7). Although pAb-Aβ recognized the characteristic small punctate capillary amyloid deposits in Tg-SwDI mice (Fig. 6d) these Aβ deposits were not appreciably labelled with mAb4G8 due to the presence of the Dutch and Iowa CAA mutations in the Aβ peptides (Fig. 6h). In contrast, both pAb-Aβ and mAb4G8 equally labelled plaque deposits in Tg2576 mice since they are composed of human wild-type Aβ (Fig. 6a,e, respectively). However, in the bigenic Tg-SwDI/Tg2576 mice both the large volume capillary amyloid deposits, as well as the parenchymal plaques, were strongly immunolabelled with both pAb-Aβ and mAb4G8 indicating that they are primarily composed of human wild-type Aβ derived from the Tg2576 background.

### Microvascular amyloid seeds Aβ with anti-parallel signature

**Figure 7:**
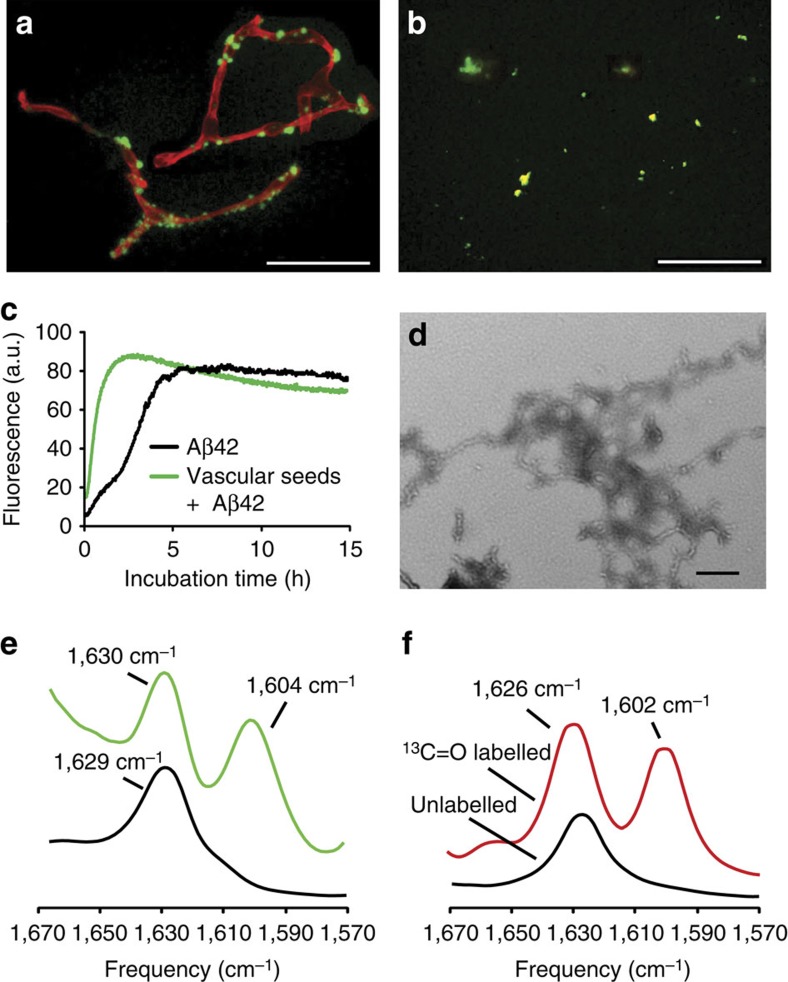
Anti-parallel β-sheet structure in vascular amyloid. Capillaries isolated from aged Tg-SwDI mouse brain (**a**) and capillary amyloid deposits after digestion and removal of the capillaries (**b**) stained for fibrillar amyloid using thioflavin S (green) and immunolabelled for cerebral blood vessels using an antibody to collagen IV (red). Scale bars, 50 μm. (**c**) Thioflavin T fluorescence showing rapid fibril growth of Aβ42 in the presence of microvascular seeds (green). (**d**) TEM of Aβ fibrils obtained by adding 1-^13^C Gly33-labelled Aβ to microvascular amyloid seeds. Scale bar, 50 nm. (**e**) FTIR spectra of Aβ fibrils formed using soluble wild-type Aβ42WT labelled with 1-^13^C Gly33 either added to seeds from microvascular amyloid deposits (green) or grown in solution without seeds (black). (**f**) FTIR spectra of KLVFFAE fibrils with (red) or without (black) 1-^13^C labelling at Leu2. (Supplementary Fig. 1). TEM, transmission electron microscopy.

In Fig. 1 and Supplementary Fig. 3 we showed *in vitro* that Aβ40-DI is metastable and can form fibrils in solution with both parallel and anti-parallel β-sheet structure, which suggests a possible difference between vascular and parenchymal amyloid since the latter has been shown to adopt a parallel β-sheet signature^26^. To address the structure of Aβ fibrils in the microvascular amyloid, we again used FTIR spectroscopy. Cerebral capillaries with amyloid deposits were isolated from aged Tg-SwDI brain (Fig. 7a) and human sporadic CAA brain (Supplementary Fig. 8), and the vascular amyloid deposits were further isolated after digestion and removal of the capillaries (Fig. 7b). The isolated vascular amyloid deposits were used as seeds. The architecture of the fibrillar seeds dictate the structure of the amyloid fibrils that assemble in their presence. The isolated seeds drove assembly of soluble wild-type Aβ42 into amyloid fibrils as assessed by the rapid increase in thioflavin T fluorescence (Fig. 7c) and a lattice of short fibrils observed by transmission electron microscopy (Fig. 7d). The fibrils have an anti-parallel β-sheet structure as defined by the intense signature FTIR absorption peak at 1,604 cm^−1^ due to labelling with 1-^13^C Gly33 (Fig. 7e, green profile). This spectrum is in contrast to the corresponding spectrum of parallel wild-type Aβ42 fibrils (also labelled with 1-^13^C Gly33) grown in solution (Fig. 7e, black profile), but remarkably similar to the spectrum of anti-parallel fibrils of the short KLVFFAE peptide (labelled with 1-^13^C Leu2) used as a control as shown in Fig. 7f (red profile) and Supplementary Fig. 1. These findings indicate that the capillary fibrillar amyloid deposits in Tg-SwDI mice contain anti-parallel β-sheet structure. In contrast, to the antiparallel fibrils formed in solution that are metastable and convert to parallel fibrils at 37 °C (Fig. 1 and Supplementary Figs 2,3), the fibrils generated from vascular amyloid fibrils were incubated at 37 °C and the signature anti-parallel band in the FTIR spectra did not change appearance over a period of months.

## Discussion

The process of misfolded proteins seeding the aberrant folding, aggregation and spreading of normal proteins is most commonly associated with the prion disorders^35^. However, this phenomenon is now recognized to occur in a number of neurodegenerative diseases including AD, Parkinson's disease, fronto-temporal dementia and amyotrophic lateral sclerosis^36^,^37^,^38^. As with prion disorders, recent work in animal models has demonstrated that misfolded protein pathology can be seeded and spread with aggregated forms of Aβ, tau and α-synuclein^36^,^37^,^38^,^39^,^40^,^41^,^42^,^43^. With regard to Aβ, both parenchymal and vascular deposits form when mice or rats expressing human AβPP are injected with exogenous Aβ seeds derived from brain tissues indicating that the seeds nucleate amyloid formation^38^,^39^,^40^,^44^,^45^. In some cases a single misfolded protein is observed in several neurodegenerative conditions, such as TDP-43 which forms aggregates in AD, amyotrophic lateral sclerosis and fronto-temporal dementia^46^. On the other hand, multiple misfolded proteins can occur in a single disorder as in AD where aggregated forms of Aβ and tau proteins are found. Another study showed that inoculation of prions in an AD transgenic mouse model accelerated and enhanced both prion and Aβ pathologies suggesting molecular crosstalk between distinct misfolded proteins^47^. In fact, it has recently been shown that like prion diseases, strains of Aβ distinguished by the differential accumulation of Aβ isoforms into distinct morphologies can be serially transmitted in mice^48^,^49^. Finally, recent findings in humans suggest that inoculation with two distinct amyloid proteins such as Aβ and PrP can promote both amyloid pathologies independently^50^.

Here we show that microvascular amyloid seeds, composed of CAA mutant forms of Aβ, can recruit normal, wild-type Aβ peptides and promote their assembly into amyloid fibrils. This observation suggests that there is molecular recognition and crosstalk between different strains of Aβ, one that exclusively forms cerebral vascular amyloid and another that produces primarily parenchymal amyloid plaques. This finding is reminiscent of previous work where it was demonstrated that one strain of prion protein formed an initial prion plaque core that could serve as a scaffold for aggregation and deposition of another strain of prion protein to form hybrid deposits in mice^51^. Similarly, in our study we show that in bigenic Tg-SwDI/Tg2576 mice early-onset capillary fibrillar amyloid deposits harbouring familial CAA mutations can serve as a scaffold to promote the fibrillar assembly and greatly enhance the accumulation of non-mutated, wild-type Aβ in cerebral capillaries. Although the present study suggests that this effect seems to be markedly enhanced with CAA mutant vascular seeds a similar process likely occurs with wild-type Aβ in sporadic CAA and AD. Indeed, previous studies have demonstrated the progression and spreading of CAA in specific vessels over time in human AβPP transgenic mice^52^,^53^.

Although the Dutch-type E22Q and Iowa-type D23N mutations in Aβ promote extensive fibrillar amyloid deposition in cerebral blood vessels there is a striking absence of parenchymal fibrillar amyloid plaques in the surrounding brain parenchyma. Although the explanation for this difference remains uncertain several experimental results regarding these specific CAA mutant Aβ peptides may provide insight into their preference for vascular accumulation. For example, both the Dutch-type (E22Q) and Iowa-type (D23N) mutations in Aβ peptide convert an acidic side chain to a polar uncharged side chain. Earlier experimental studies demonstrated that both of these mutations in Aβ can markedly enhance amyloid fibril assembly and the cytotoxic properties of the peptide in comparison with non-mutated Aβ (refs 29, 54, 55, 56, 57). Also, previous studies showed that cultured cerebrovascular cells and isolated cerebral vessels contain high levels of GM3 ganglioside, a form that selectively stimulates fibrillar formation of CAA mutant types of Aβ (refs 58, 59). Further, forms of Aβ with CAA mutations were found to be poorly cleared from brain, either through cerebrospinal fluid or across the blood–brain barrier into the periphery^29^,^60^. This reduced clearance of CAA mutant Aβ peptides could significantly contribute to their pathologic accumulation in cerebral blood vessels. Lastly, CAA mutations in Aβ may cause profound structural changes in the conformation of the peptides that lead to selective assembly and deposition in cerebral vessels rather than parenchymal plaques. In this regard, it was shown that under certain experimental conditions the D23N Iowa CAA mutant form of Aβ can assemble into fibrils with an anti-parallel structure, which differs from the parallel fibril signature that is more commonly seen with non-mutated Aβ peptides^27^,^28^. Similarly, we show that the Dutch/Iowa mutant form of Aβ can also form anti-parallel amyloid fibrils (Fig. 1). Indeed, we further show that isolated cerebral capillary amyloid seeds from Tg-SwDI mice promote strong anti-parallel fibril assembly of wild-type Aβ (Fig. 7). Further, this vascular anti-parallel fibril configuration is not restricted to mice or to CAA mutant amyloid as cerebral vascular amyloid extracted from a human sporadic CAA case, composed of non-mutated Aβ, similarly drove anti-parallel amyloid fibril formation (Supplementary Fig. 8). The anti-parallel structure may predispose soluble seeds to accumulate on cerebral blood vessels. It is likely that a combination of these altered properties related to CAA mutant forms of Aβ, coupled together with age-related changes to cerebral vessels and basement membrane components^61^, contribute to its selective and robust accumulation as anti-parallel, fibrillar deposits in the cerebral vasculature.

Although the CAA mutant forms of Aβ exhibit these altered properties they exist in the presence of wild-type Aβ peptides in the brain since familial CAA patients generally harbour one CAA mutant and wild-type *APP* allele^7^,^8^,^10^. However, fibrillar amyloid is primarily restricted to the cerebral vessels in CAA patients with the absence of appreciable parenchymal plaque amyloid^9^,^10^. Although CAA mutant forms of Aβ may promote cerebrovascular amyloid for the reasons mentioned above, it does not explain the absence of parenchymal plaque amyloid, even though wild-type Aβ peptides are still produced. As suggested above, CAA mutant Aβ peptides or small soluble oligomers may impart an altered anti-parallel conformation on the co-existing wild-type Aβ peptides that is amenable to vascular, but not parenchymal, amyloid formation. In addition, the structure of the Aβ42 peptide in parenchymal amyloid may be distinctly different than the structure that either CAA mutant Aβ40 or wild-type Aβ40 can adopt (see Supplementary Fig. 9). In any case, our studies here show that highly fibrillogenic CAA mutant forms of Aβ can strongly drive the assembly and cerebral vascular deposition of wild-type Aβ with an anti-parallel signature. Further elucidating the distinct structural elements of CAA mutant and wild-type Aβ peptide assemblies may provide additional insight into the unique pathology of CAA.

## Methods

### Peptide synthesis

Aβ peptides were synthesized using tBOC-chemistry (Keck Large Scale Peptide Synthesis Facility, Yale University) and purified by high-performance liquid chromatography using linear water−acetonitrile gradients containing 0.1% (v/v) trifluoroacetic acid. The mass of the purified peptide was measured using matrix-assisted laser desorption or electrospray ionization mass spectrometry, and was consistent with the calculated mass for the peptide. On the basis of analytical reverse phase high-performance liquid chromatography and mass spectrometry, the purity of the peptides was 95–99%.

### Peptide preparation

Monomeric Aβ peptide was prepared by first dissolving purified peptides in 1,1,1,3,3,3-hexafluoro-2-propanol, flash freezing in liquid nitrogen and then lyophilizing under a 25 mTorr vacuum overnight. Lyophilized Aβ peptides were dissolved in a small volume of 50 mM NaOH, diluted in low-salt buffer (10 mM phosphate buffer, 10 mM NaCl, pH 7.4) at 4 °C and then filtered with 0.2 μm filters before use.

### FTIR spectroscopy

The FTIR spectra were obtained from 400–4,000 cm^−1^ on a Bruker IFS 66V/S spectrometer equipped with a liquid N_2_-cooled mercury cadmium telluride detector. Samples were prepared by drying a 50–100 μl of peptide solution on the surface of a germanium ATR plate. To distinguish, parallel and antiparallel β-sheet structure the peptides were labelled at residue 33 with 1-^13^C glycine (Supplementary Figs 2 and 3). The ^13^C=O label results in a splitting of the 1,630 cm^−1^ β-sheet resonance and the appearance of an intense IR band between ∼1,600 and 1,610 cm^−1^ (refs 26, 27). Anti-parallel β-sheet Aβ40-DI fibrils and KLVFFAE fibrils were prepared using the methods described previously^62^ and in Supplementary Fig. 1. For the preparation of fibrils from isolated vascular amyloid, isolated vascular amyloid was sonicated for 30 min and then mixed with 10 μM Aβ42 using a 5:195 or 10:190 volume of seed material to 10 μM Aβ42 stock, and incubated at 37 °C.

### Thioflavin T measurements

All Aβ peptides were solubilized in 100 mM NaOH and diluted in 10 mM phosphate buffer to a concentration of 100–200 μM at pH 7.4. For seeding experiments solutions of Aβ40-DI were prepared as above and incubated at 37 °C under agitating conditions for 1 week, before addition as seeds. Fresh solutions of wild-type Aβ40 and Aβ42 were prepared as above and added to make a final peptide solution that was 90% wild-type Aβ40 or Aβ42 and 10% Aβ40-DI seeds. Thioflavin T fluorescence measurements were performed on a Horiba Jobin Yvon Fluorolog FL3-22 or SpectraMax M2 (with plate reader) spectrofluorimeter. Samples were excited at a wavelength of 461 nm and fluorescence emission spectra were collected from 475 to 550 nm. At the conclusion of the experiments the assembly of amyloid fibrils was confirmed by transmission electron microscopy.

### Transmission electron microscopy

Samples were diluted, deposited onto carbon-coated copper mesh grids and negatively stained with 2% (w/v) uranyl acetate. The excess stain was wicked away, and the sample grids were allowed to air dry. The samples were viewed with an FEI Tecnai 12 BioTwin 85 kV transmission electron microscope, and digital images were taken with an Advanced Microscopy Techniques camera.

### Animals

Generation and characterization of Tg-SwDI mice were previously described^28^,^29^,^31^. Tg2576 mice were generated as previously described^30^ and obtained from the Jackson Laboratories. Heterozyous Tg-SwDI mice on a pure C57/Bl6 background were bred with heterozygous Tg2576 mice on a mixed C57/Bl6/Sjl/129 background to obtain heterozygous Tg-SwDI, heterozygous Tg2576, and bigenic Tg-SwDI/Tg2576 mice all on the same mixed background. On the basis of our previous experience with measuring pathology in each transgenic line an *n*=10–12 mice of each genotype were examined at 6, 12 and 18 months of age for quantitative immunochemical and pathological studies. In this study, mice were included solely based on genotype and groups were composed of a mixture of males and females. All work with animals was approved by the Stony Brook University Institutional Animal Care and Use Committee and in compliance with the guidelines established by the Public Health Service Guide for the Care and Use of Laboratory Animals.

### Intra-hippocampal injection of Aβ peptides in mice

Twelve months old Tg-SwDI were anesthetized with i.p. injections of ketamine/xylazine (100 mg kg^−1^ /10 mg kg^−1^) and positioned in a stereotaxic frame (KOPF Instruments, Tujunga, CA, USA). A sagittal incision was made caudal to rostral allowing the scalp to be retracted and held in place with micro-clips to expose the skull surface. To insert the Hamilton syringe (30 gauge), a small burr hole was drilled in the parietal bone. Two microlitre of freshly prepared biotin-labelled wild-type Aβ40 or biotin-labelled wild-type Aβ42 peptide (0.5 mg ml^−1^, dissolved in 0.9% NaCl) was injected stereotactically into the hippocampus (bregma—2.00 mm anterior, lateral—1.50 mm, depth—1.80 mm) at a rate of 0.3 μl min^−1^ using a micro-injection unit. The needle was left *in situ* for 5 min post injection to minimize reflux before being slowly removed. The scalp was closed under sterile conditions using a 4-0 nylon suture. The mice were placed in a cage warmed with a heating pad and observed until alert and exhibiting normal behaviour. Twenty-four hours post injection the mice were killed and the brains were collected for immunohistochemical analysis.

### Immunohistochemical analysis

Mice were killed at designated time points. The brains were immediately removed and bisected in the mid-sagittal plane. One hemisphere was snap-frozen and used for the protein analyses. The other hemisphere was placed in 70% ethanol, followed by xylene treatment and embedding in paraffin. For immunohistochemistry and histology brain sections were cut in the sagittal plane at 10 μm thickness using a microtome, deparaffinated and rehydrated. Antigen retrieval was performed by treatment with proteinase K (0.2 mg ml^−1^) for 10 min at 22 °C for Aβ and collagen staining. Primary antibodies were detected with horseradish peroxidase-conjugated or alkaline phosphatase-conjugated secondary antibodies and visualized either with a stable diaminobenzidine solution (Invitrogen, Carlsbad, CA) or with the fast red substrate system (Spring Bioscience, Fremont, CA), respectively, as substrate. Sections were counterstained with haematoxylin. Alternatively, primary antibodies were detected with Alexa Fluor 594-conjugated donkey anti-rabbit or Alexa Fluor 488-conjugated goat anti-mouse secondary antibodies (1:1,000). Staining for fibrillar amyloid was performed either using Amylo-Glo as described by the manufacturer (Biosensis Inc., Thebarton, South Australia) or using thioflavin S^63^. The following antibodies were used for immunohistochemical analysis: rabbit polyclonal antibody to Aβ (pAb-Aβ), which recognizes amino terminal residues 1-5 of human Aβ (ref. 33) (1:200), mouse monoclonal 4G8, which recognizes an epitope between residues 17–24 of wild-type Aβ (ref. 32) (1:200), rabbit pAb to Aβ40 (1:500), mAb to Aβ42 (1:1,000) and rabbit polyclonal antibody to collagen type IV (1:100, Research Diagnostics Inc., Flanders, NJ).

### ELISA measurement of Aβ peptides

Soluble pools of Aβ40 and Aβ42 were determined by using specific ELISAs on carbonate-extracted mouse forebrain tissue and subsequently the insoluble Aβ40 and Aβ42 levels were determined by ELISA of guanidine lysates of the insoluble pellets resulting from the carbonate extracted brain tissue^64^,^65^. Total Aβ40 and Aβ42 levels were determined by combining the soluble and insoluble pools of each species. In the sandwich ELISAs Aβ40 and Aβ42 were captured using their respective carboxyl-terminal-specific antibodies m2G3 and m21F12 and biotinylated m3D6, specific for human Aβ, was used for detection. Each brain lysate was measured in triplicate and compared to linear standard curves generated with known concentrations of human Aβ using a Spectramax M2 plate reader (Molecular Devices, Sunnyvale, CA).

### Quantitation of parenchymal and capillary amyloid load

The area of thioflavin-S labelled parenchymal amyloid plaques and capillaries in the regions of the subiculum, thalamus and fronto-temporal cortex was respectively quantified on the same set of systematically sampled thioflavin-S stained sections using stereological principles^66^. To determine capillary CAA volume 50 affected capillaries were randomly chosen in specific brain regions from 18-month-old mice of each genotype. The actual capillary amyloid volume was determined after subtracting the volume of the capillary harbouring the amyloid deposits.

### Isolation of cerebral capillary amyloid seeds

Brain capillaries were isolated from the cortices of twelve to eighteen Tg-SwDI mice and homogenized in TBS using a Kontes glass dounce homogenizer. The homogenates were centrifuged at 16,000*g* for 5 min and the top aqueous layer was removed and discarded. The pellets were washed once recentrifuged and then re-suspended in a solution of 17% Dextran/TBS (MW 65,000–85,000). The samples were re-homogenized in the dounce homogenizer and centrifuged at 10,000*g* for 10 min. The top lipid layer and supernatant were removed and the vessel pellet was washed 2 × with TBS, centrifuged 10,000*g* for 5 min, resuspended in TBS, passed through a 40 μm filter and washed several times with TBS. The vessels were washed off the filter with TBS and collected by centrifugation at 16,000*g* for 5 min. The concentrated microvessel pellet was resuspended in 500 μl of TBS, applied to a gradient prepared using 22, 19 and 15% Optiprep (Sigma, St Louis, MO) in TBS and centrifuged at 6,000*g* for 20 min. The resulting pellet containing the microvessels was washed twice with TBS and centrifugation. The resulting microvessel pellet was resuspended in 100 μl of TBS and an aliquot was analysed microscopically by staining with thioflavin S and immunolabelling with an antibody to collagen IV to confirm the isolation of amyloid-containing microvessels. The remaining microvessels were treated with 3 mg ml^−1^ collagenase at 37 °C overnight. The collagenase treated samples were centrifuged at 16,000*g* for 5 min and the resulting pellet was washed twice with TBS. The amyloid pellet was resuspended in TBS and again analysed microscopically by staining with thioflavin S and immunolabelling with an antibody to collagen IV to confirm the isolation of vascular amyloid deposits and the absence of microvessels.

### Statistical analysis

In the pathology experiments the statistical differences between pairs of data sets were analysed by a two-tailed unpaired *t*-test and presented with standard deviations. Values of *P*<0.05 were considered statistically significant. Statistical analysis was done with the Prism 5.0 program (GraphPad).

### Data availability

The data that support the findings of this study are available from the corresponding author upon reasonable request.

## Additional information

**How to cite this article:** Xu, F. *et al*. Cerebral vascular amyloid seeds drive amyloid β-protein fibril assembly with a distinct anti-parallel structure. *Nat. Commun.* **7,** 13527 doi: 10.1038/ncomms13527 (2016).

**Publisher's note:** Springer Nature remains neutral with regard to jurisdictional claims in published maps and institutional affiliations.

## Supplementary Material

Supplementary Information Supplementary Figures 1-9 and Supplementary References
