# Author Correction: Cerebral vascular amyloid seeds drive amyloid β-protein fibril assembly with a distinct anti-parallel structure

**DOI:** 10.1038/s41467-024-50272-6

**Published:** 2024-07-19

**Authors:** Feng Xu, Ziao Fu, Sharmila Dass, AnnMarie E. Kotarba, Judianne Davis, Steven O. Smith, William E. Van Nostrand

**Affiliations:** 1 Departments of Neurosurgery and Medicine, Stony Brook University, Stony Brook, New York 11794 USA; 2 Department of Biochemistry and Cell Biology, Stony Brook University, Stony Brook, New York 11794 USA

Correction to: *Nature Communications* 10.1038/ncomms13527, published online 21 November 2016

The original version of this Article contained an error in Fig. 4a and Fig. 4i, and in the figure legend for Fig. 4.

In Fig. 4a, the representative image in this panel showing amyloid pathology in Tg-SwDI mice at 6 months of age was inadvertently duplicated from Fig. 4 of a previous publication by the same group^1^.

In Fig. 4i, the representative image of capillaries with enlarged amyloid deposits that were observed in 18 months old bigenic Tg-SwDI/Tg2576 mice was an aggregate of representative amyloid laden vessels from three different brain regions used for the quantitative measures presented in panels (j–l) of Figure 4, but this was not demarcated on the figure panel. The representative vessels presented in the original composite image in Fig. 4i were not used for the quantitative comparisons of percentage Aβ immune-positive capillaries (Fig. 4k) nor in measures of capillary amyloid volume (Fig. 4l) between the three brain regions.

The correct version of Fig. 4, with a separate representative image of amyloid pathology in Tg-SwDI mice at 6 months of age in Fig 4a, and appropriate demarcation of the three vessels and identified which brain region each vessel in Fig 4i, is shown here:
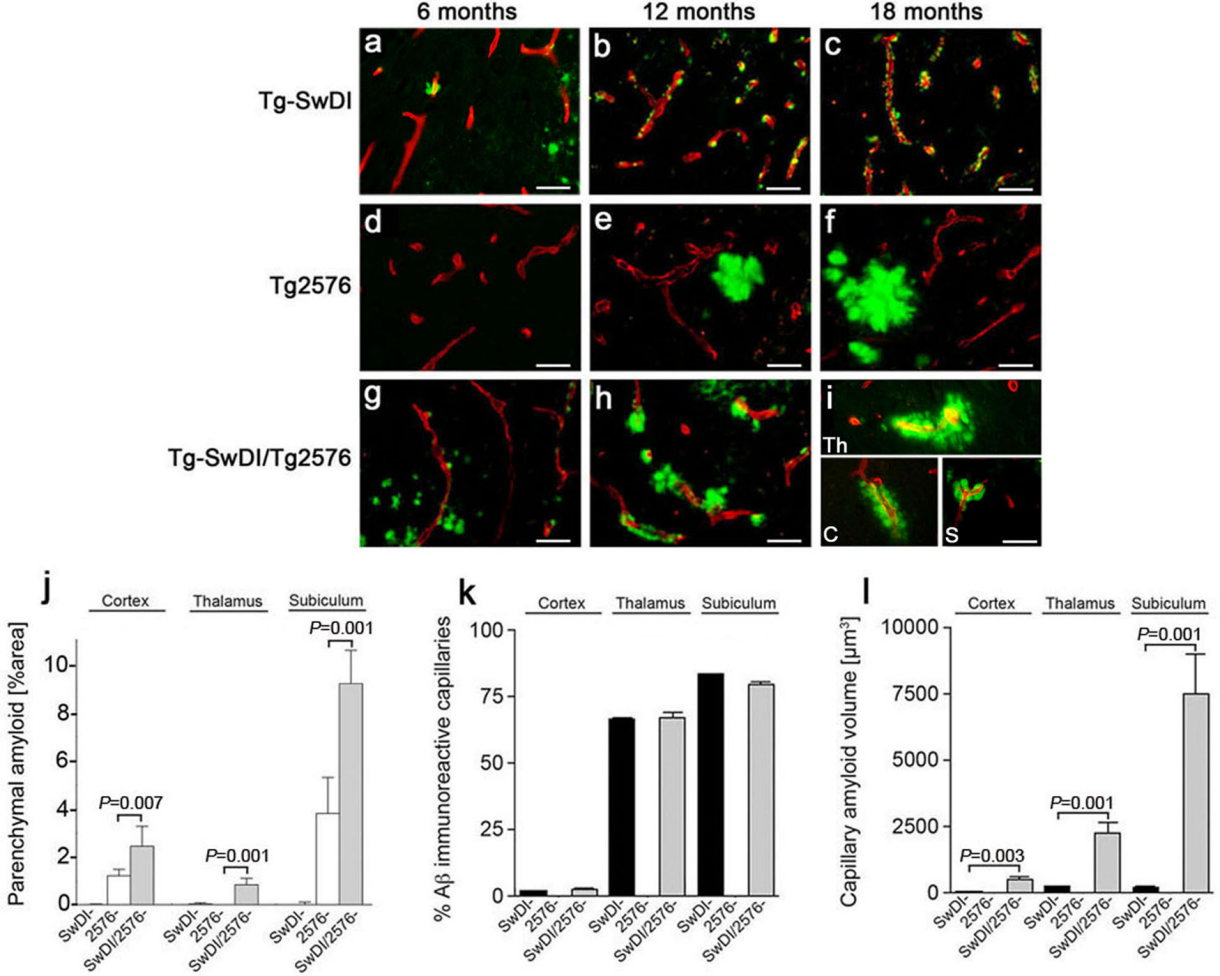


The incorrect version of Fig. 4 is here:
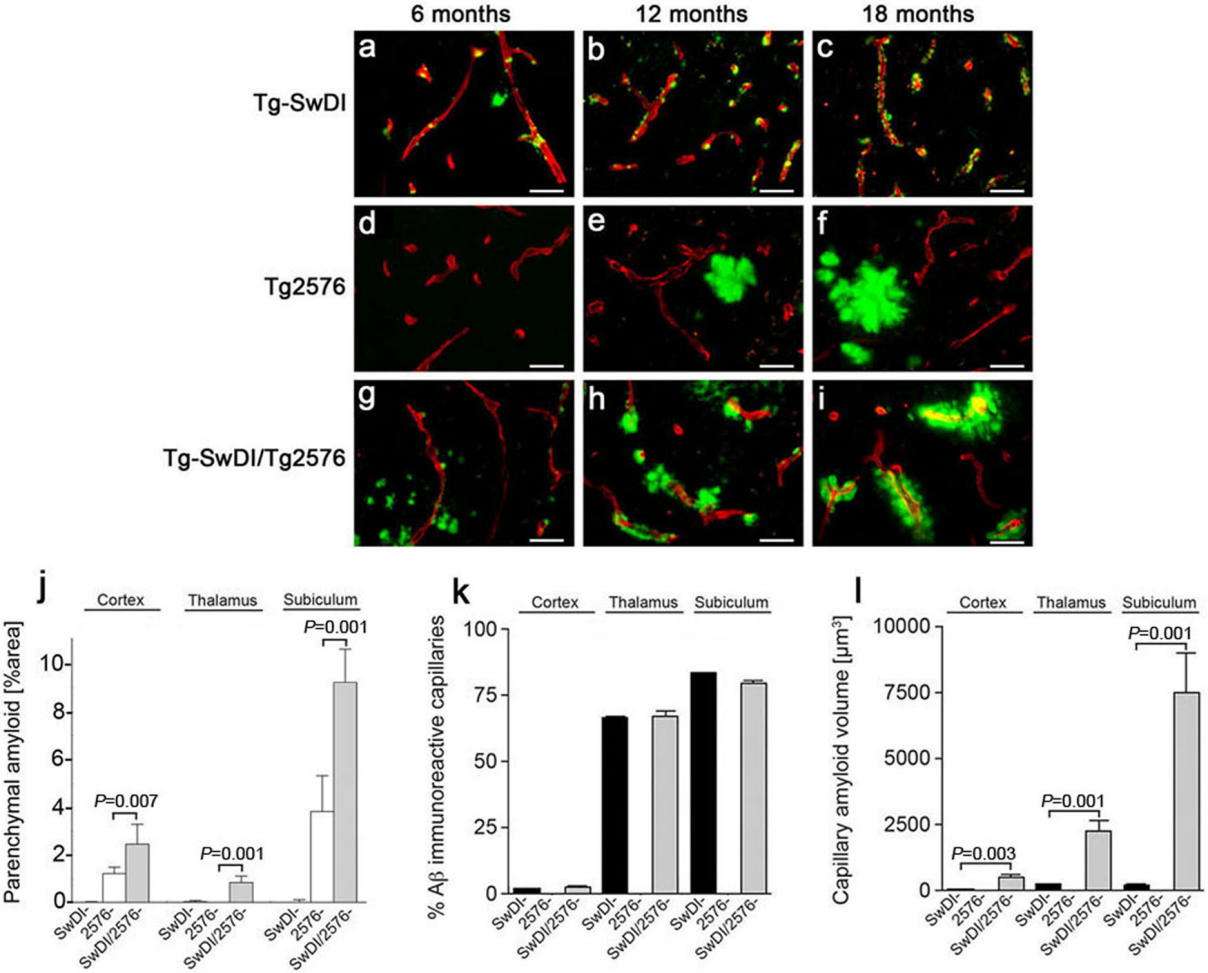


In addition, the correct figure legend for Figure 4 reads ‘(i) Composite image showing individual amyloid laden capillaries from the cortex (C), thalamus (Th), and subiculum (S) in the bigenic animals at 18 months.’ after ‘(g–i) bigenic Tg-SwDI/Tg2576 mice’.
