# Supplementary material for: Cerebral vascular amyloid seeds drive amyloid β-protein fibril assembly with a distinct anti-parallel structure
Source: Nat Commun. 2016 Nov 21;7:13527. doi: 10.1038/ncomms13527 (PMC5121328; doi:10.1038/ncomms13527)
Supplement: Supplementary Information — Supplementary Figures 1-9 and Supplementary References [file ncomms13527-s1.pdf]

Supplementary Figure 1

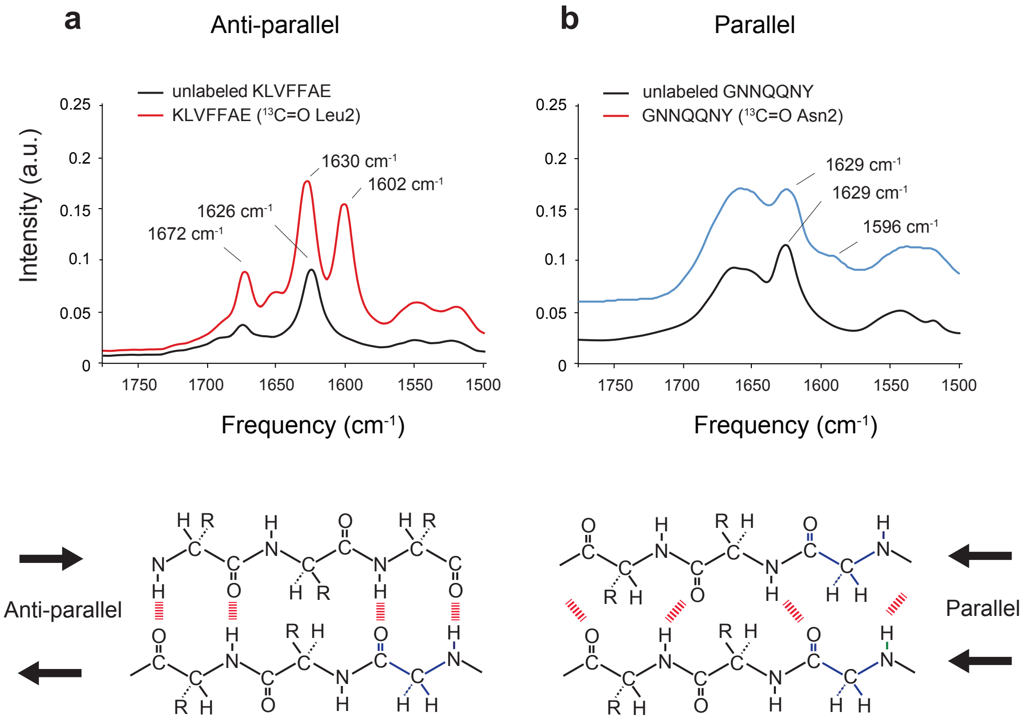

Supplementary Figure 1. FTIR of parallel and anti-parallel fibril model compounds. FTIR spectroscopy is a well-established structural probe[1]. The frequency of the amide I vibration, which lies between 1600 and 1700 cm^{-1}, is diagnostic of protein secondary structure. Helical secondary structure typically exhibits a narrow band at ~1650-1655 cm^{-1}, while β-sheet typically exhibits a band at 1630 cm^{-1}. These correlations were originally derived from infrared studies of proteins containing known secondary structural elements. Recently, isotope-edited FTIR studies have provided a way to distinquish β-sheets with either anti-parallel or parallel β-strands[2,3]. Here, we illustrate the differences exhibited in FTIR spectra of these two types of β-sheet structure using model compounds that have previously been characterized as having anti-parallel or parallel β-strands.

(**a**) The seven residue KLVFFAE peptide forms fibrils with anti-parallel β-sheet structure. This peptide corresponds to residues 16-22 of Aβ40. The anti-parallel structure, which was established by solid state NMR spectroscopy[4], is likely driven by the complementary electrostatic interactions of the N- and C-termini of the peptide when the β-strands associate in anti-parallel arrangement. In panel (**a**) we show the amide I

region of Aβ16-22 (red) containing 1-^{13}C labeled Leu2 (corresponding to Leu17 in the Aβ40 sequence) and unlabeled Aβ16-22 (black). Fibrils were harvested after incubating at 37 °C for 10 days. The 1626 cm^{-1} resonance in the spectrum of the unlabeled peptide is characteristic of β-sheet secondary structure. The splitting into two intense bands at 1602 and 1630 cm^{-1} is associated with anti-parallel β-sheet [2,3]. a.u. = arbitrary units.

(**b**) The seven-residue GNNQQNY peptide, a fragment of the yeast prion protein Sup35p, forms fibrils with parallel, in-register β-strands. The parallel, in-register structure in this peptide, which was also established by solid-state NMR measurements [5] is likely driven by hydrogen bonding interactions between the side chain amide groups of Asn and Gln. In this case, isotope labeling does not shift the major symmetric 1629 cm^{-1} band but results in a weak isotope shifted resonance at 1596 cm^{-1}. The broad intensity at ~1650 cm^{-1} is attributed to the side chain Asn and Gln amide vibrations.

Molecular structures of the parallel and anti-parallel β-strands are shown below the FTIR spectra to illustrate that the hydrogen bonding arrangement is different in the two geometries. This difference in hydrogen-bonding gives rise to the differences in the amide I vibration, which corresponds largely to the C=O stretching vibration.

The differences in the vibrational spectra for anti-parallel and parallel β-strands within β-sheet secondary structure observed for these two model peptides are the same those found by vibrational calculations on anti-parallel and parallel β-sheets[2]. Namely, for anti-parallel structure the major component of the amide I normal mode splits into two equally intense bands (one with higher frequency and one with lower frequency). In contrast, for parallel structure the major β-sheet peak does not change frequency or intensity, but a very weak isotope shifted band is observed at a lower frequency than the intense low-frequency component of the amide I band observed in the spectra of the anti-parallel structure. The differences arise in how the internal coordinates couple within the amide I normal mode. This coupling is dependent on the geometry.

Supplementary Figure 2

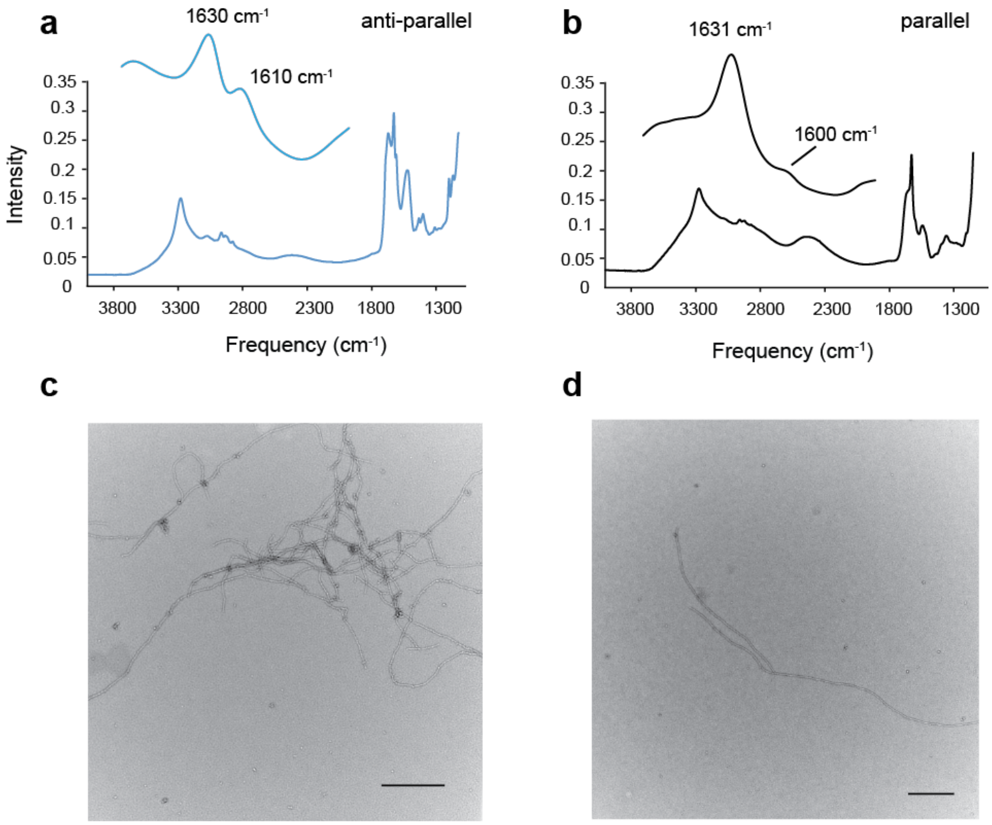

Supplementary Figure 2. Aβ40-DI fibrils with parallel and anti-parallel structure. FTIR spectroscopy of Aβ40-DI containing 1-^{13}C-Gly33 provides a probe for anti-parallel β-sheet secondary structure. Here we describe the protocol for obtaining both anti-parallel and parallel fibrils of Aβ40-DI, and compare FTIR spectra and TEM images of the resulting fibrils.

(**a**) FTIR spectrum of Aβ40-DI with anti-parallel structure. Inset shows the amide I region (see also Supplementary Fig. 3). The anti-parallel fibrils of Aβ40-DI were obtained using the following procedure modified from that of Tycko and colleagues[1]. Pure Aβ40-DI peptide was dissolved in DMSO at a concentration of 2 mM (~10 mgs in 0.5 mL DMSO). A 100 μL aliquot was taken and diluted into 5 mL 10 mM phosphate buffer (10 mM NaCl) such that the final concentration of DMSO was 2%, and the concentration of Aβ40-DI was 100 μM. The solution was filtered with a 0.22 μM filter and allowed to fibrillize overnight. The solution was then transferred into a glass vial and bath sonicated (15 min). Another 5 mL of cold phosphate buffer was added to the sonicated

solution along with another 100 microliters of the DMSO stock solution of Aβ40-DI. This material was allowed to fibrillize for 3 h in the cold room and then filtered twice with 0.2 μM filters. Another 100 microliters of DMSO stock in 5 mL of cold phosphate buffer was added to the filtered solution. Comparison of the amide 1 region (1600-1700 cm^{-1}) of the spectrum obtained at 22 °C after incubating for 1 week in panel (**a**) with the spectrum obtained of fibrils immediately following the anti-parallel fibril preparation at 4-6 °C (see Supplementary Fig. 3a) shows there is considerably more random coil structure in the sample prepared and kept at low temperature. The high frequency region of the FTIR spectrum (~3000 cm^{-1}) contains water vibrations and indicates that the samples contain comparable amounts of residual hydration. (**b**) FTIR spectrum of Aβ40-DI with parallel β-sheet structure obtained by incubation at 37 °C. (**c**) TEM images of antiparallel fibrils at 22 °C. Scale bar = 100 nm. (**d**) TEM images of parallel fibrils obtained by incubating Aβ40-DI at 37 °C. The anti-parallel fibrils have a more curved appearance than the parallel fibrils as described by Tycko and coworkers[6]. Scale bar = 100 nm.

Supplementary Figure 3

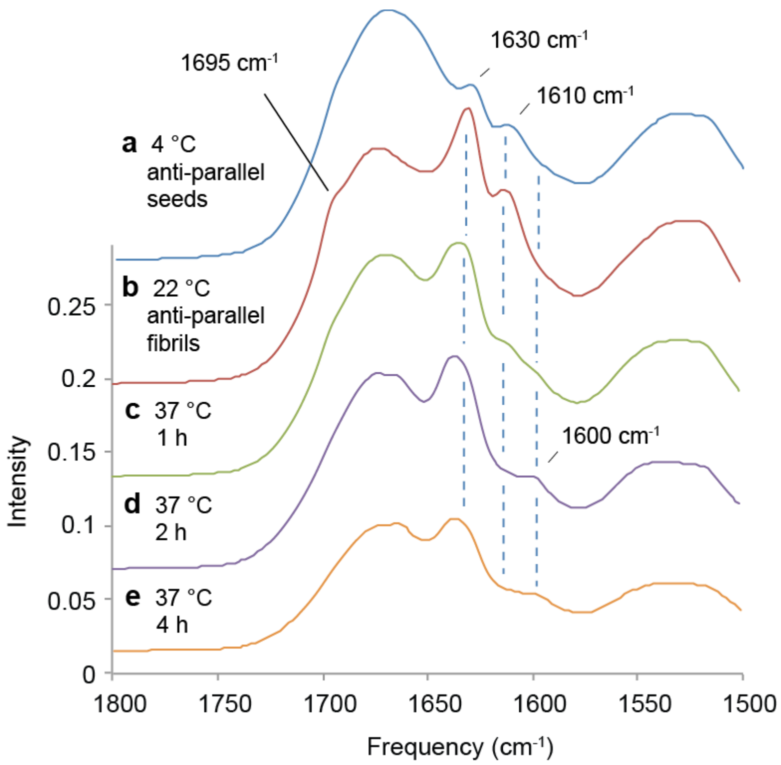

Supplementary Figure 3. Anti-parallel fibrils of Aβ40-DI formed in solution are meta-stable. Anti-parallel fibrils of Aβ40-I were previously found to be meta-stable[6]. Here we show that for anti-parallel fibrils of Aβ40-DI we observe a conversion to parallel structure upon increasing the temperature from 4 °C to 37 °C. (**a**) FTIR spectra of the anti-parallel seeds prepared at 4-6 °C using the protocol described in Supplementary Fig. 2. (**b**) Increasing the temperature to 22 °C leads to the formation of fibrils as observed by TEM (Fig. 1b). The FTIR spectrum does not change over a week of incubation at this temperature. (**c-e**) Increasing the temperature to 37 °C leads to a conversion of the anti-parallel fibrils to parallel fibrils. The change is detected by the shift (and loss of intensity) of the 1610 cm^{-1} band to 1600 cm^{-1}. This conversion indicates that the anti-parallel fibrils are meta-stable.

AFM images of the Aβ40-D and Aβ40-I peptides (unpublished) indicate that the anti-parallel fibrils are composed of laterally associated oligomers as observed for Aβ42[2]. We suggest that the lower intensity of the ~1604-1610 cm^{-1} band in the *in vitro* experiments compared to the seeded fibrils is due to a mixture of both parallel and anti-parallel ß-strands within the "protofibrils" formed *in vitro*. In contrast to the fibrils, the

fibrils seeded from vascular amyloid are stable at 37 °C. The anti-parallel peak observed in Fig. 7e (main text) is more intense than observed above and does not change upon incubation at 37 °C.

Supplementary Figure 4

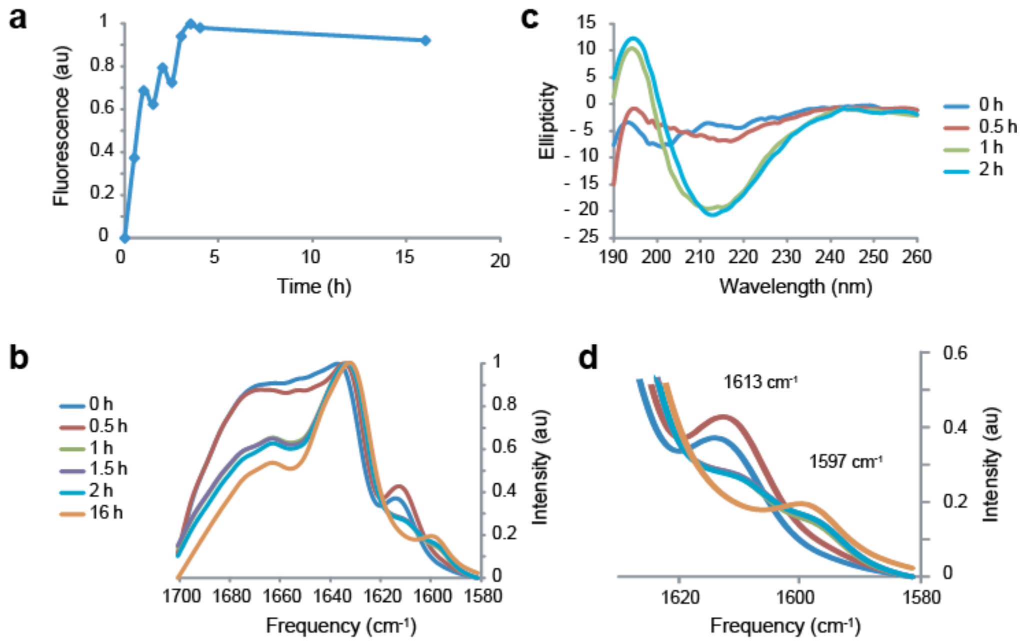

Supplementary Figure 4. Transient anti-parallel structure occurs in the formation of parallel Aβ40-DI fibrils. Intramolecular antiparallel β-strands can be found within the Aβ peptide due to the formation of β-hairpin secondary structure in pre-fibrillar intermediates[7] and intermolecular antiparallel β-strands can occur within the cross β-sheets of fibrils[6]. We have recently shown that β-hairpin secondary structure develops within the high MW oligomers and protofibrils of Aβ42 during the conversion to mature fibrils[1]. In this supporting figure, we show for Aβ40-DI that transient anti-parallel structure also occurs prior to the formation of stable β-sheet secondary structure (i.e. before the formation of mature fibrils). This transient appearance of anti-parallel structure in *non-fibrillar intermediates* is distinct from the anti-parallel structure observed in the *fibrils* generated from vascular amyloid seeds.

(**a**) Aβ40-DI forms fibrils rapidly in solution. Thioflavin T measurements show there is a rapid rise fluorescence associated with fibril formation at 37 °C starting from solutions of monomeric Aβ40-DI (100 μM). The halfway point in the transition occurs at ~0.5 h and the transition is largely complete by 3-4 hours. (**b**) Formation of β-sheet secondary structure accompanies fibril formation. Circular dichroism spectra were obtained of the Aβ40-DI peptide as a function of incubation time. The Aβ40-DI peptide is largely random

coil before 1 h and converts to β-sheet secondary structure after ~1 h. At 37 °C, Aβ40-DI forms β-sheet structure in which the β-strands have a parallel orientation (Supplementary Fig. 2). (**c**) FTIR spectra of Aβ40-DI containing 1-^{13}C Ile31 and 1-^{13}C Val39 obtained as a function of incubation time reveal several features. The spectra obtained at 0 h and 0.5 h exhibit an isotope-shifted resonance at 1613 cm^{-1} characteristic of antiparallel structure. This band losses intensity and shifts to 1597 cm^{-1} as fibrils form. (**d**) Expansion of the FTIR spectra in the region of 1580-1630 cm^{-1} showing the amide I vibrational bands associated with the conversion of ant-parallel β-hairpin structure (observed prior to fibril formation) to β-sheet with parallel β-strands (after fibril formation).

Supplementary Figure 5

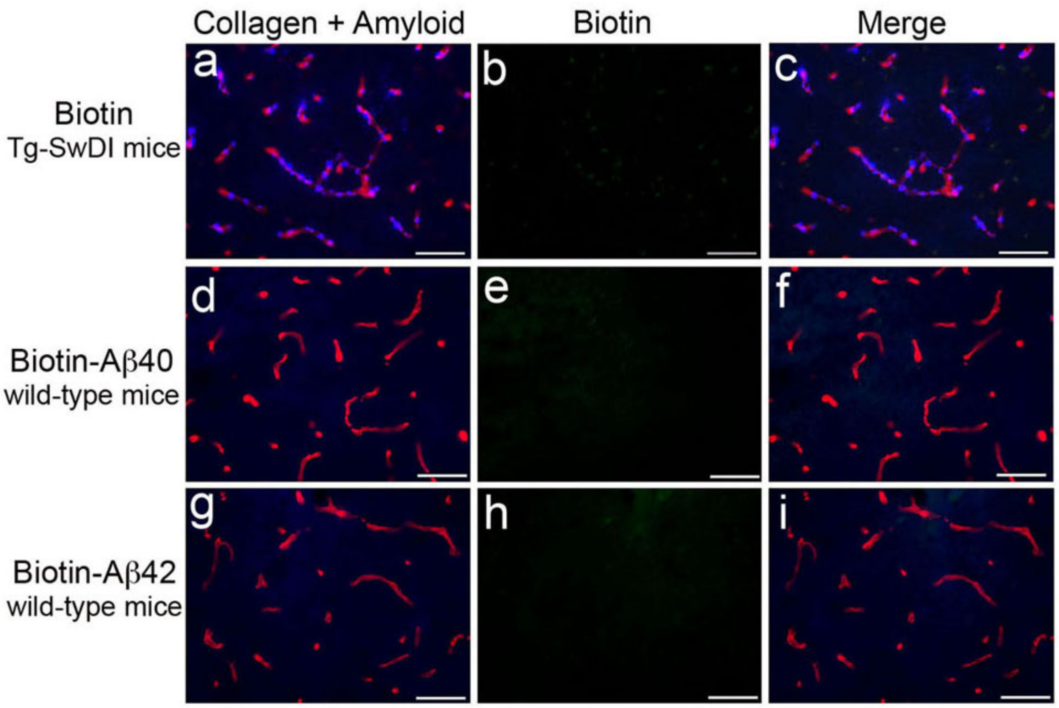

Supplementary Figure 5. Dutch/Iowa CAA mutant cerebral microvascular amyloid in Tg-SwDI mice does not accumulate administered biotin and aged wild-type mice do not accumulate biotin-labeled wild-type Aβ peptides. In this supporting figure, we show that unlike biotin-labeled wild-type Aβ peptides, intrahippocampal administered biotin alone does not accumulate on pre-exisiting cerebral capillary amyloid deposits in Tg-SwDI mice. Further, we show that intrahippocampal administered biotin-labeled wild-type Aβ peptides do not accumulate on cerebral capillaries of aged wild-type mice that lack vascular amyloid deposits.

Biotin was injected into the hippocampal region of twelve months old Tg-SwDI mice (**a-c**). Alternatively, biotin-labeled wild-type Aβ40 (**d-f**) or biotin-labeled wild-type Aβ42 (**g-i**) was injected into the hippocampus of similarly aged wild-type mice lacking capillary amyloid deposits. Brain sections were prepared and fibrillar amyloid was detected using Amylo-Glo (blue) and immunolabeled with an antibody to collagen IV for detection of cerebral blood vessels using Alexa Fluor 594-conjugated donkey anti-rabbit (red). Biotin or biotin-labeled wild-type Aβ40 or Aβ42 peptides were detected using streptavidin-Alexa Fluor 488 (green). Scale bars = 50 μm.

Supplementary Figure 6

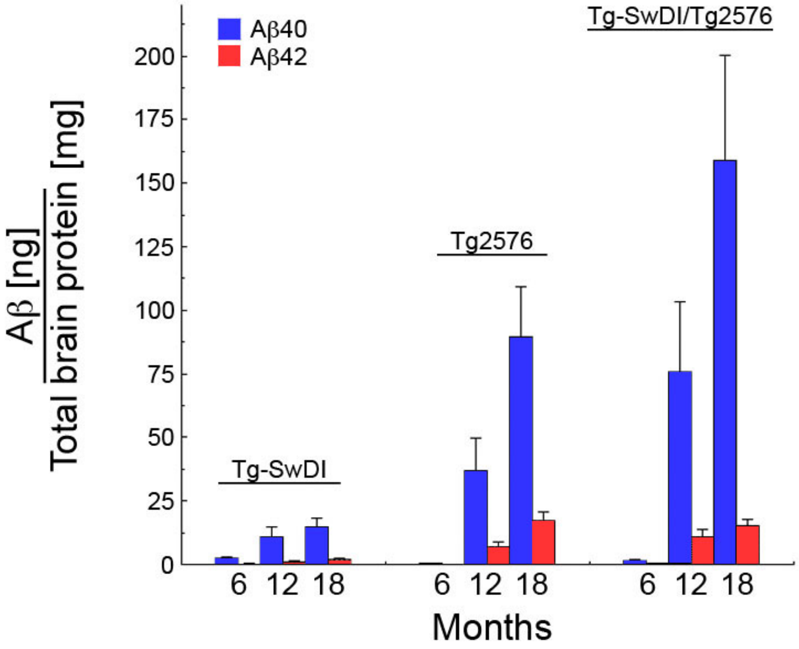

Supplementary Figure 6. ELISA measurement of cerebral human Aβ40 and Aβ42 levels in Tg-SwDI, Tg2576 and bigenic Tg-SwDI/Tg2576 mice. In this supporting figure, we show that there is a progressive accumulation of both Aβ40 and Aβ42 in each single transgenic and in the bigenic mice. Further, the data show that accumulation of Aβ40 in the bigenic mice exceeds the additive levels of Aβ40 in the single Tg-SwDI and Tg2576 lines.

The total levels of Aβ40 (blue bars) and Aβ42 (red bars) were determined in mouse forebrain extracts of six to eighteen month old mice as described in Methods. The data presented are the mean ± S.D. of 10-12 mice per time point.

Supplementary Figure 7

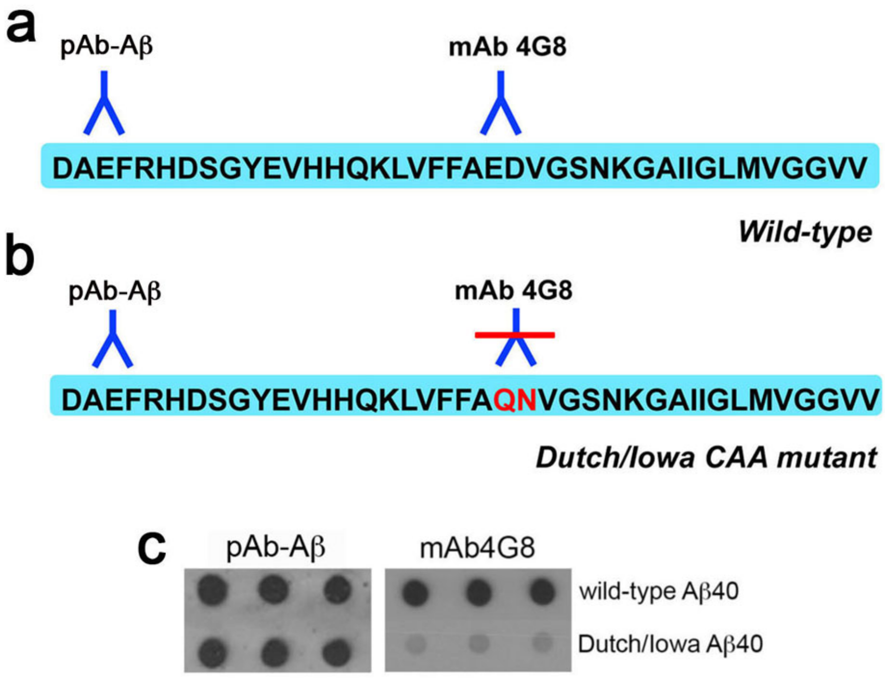

Supplementary Figure 7. Recognition of CAA mutant and wild-type Aβ peptides by pAb-Aβ and mAb4G8. (**a**) Wild-type human Aβ peptides are recognized by both the N-terminal pAb-Aβ and mid-region mAb4G8. (**b**) CAA mutant human Aβ peptides are recognized by the N-terminal pAb-Aβ but are not recognized by mid-region mAb4G8 due to the presence of the E22Q, D23N Dutch and Iowa mutations. (**c**) Dot blot analysis confirming that pAb-Aβ recognizes both wild-type and CAA mutant Aβ40 whereas mAb4G8 only recognizes wild-type Aβ40.

Supplementary Figure 8

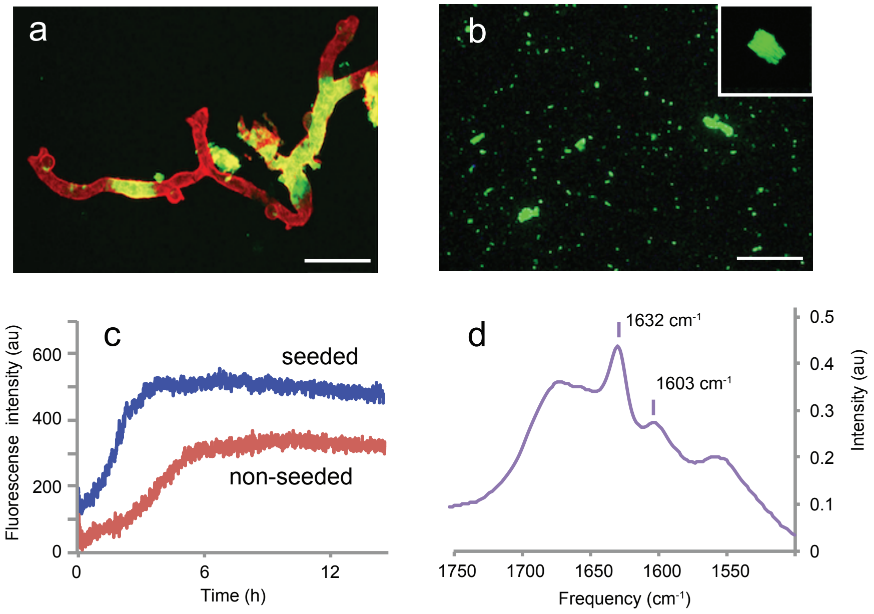

Supplementary Figure 8. Amyloid fibrils obtained from seeds of human sporadic vascular amyloid. The studies described in the main text set the stage for parallel studies on vascular amyloid derived from human brain associated with sporadic CAA or associated with CAA resulting from the Aβ40-D or Aβ40-I mutations. (**a**) Microvessels isolated from human brain associated with sporadic CAA stained for fibrillar amyloid using thioflavin S (green) and immunolabeled for cerebral blood vessels using an antibody to collagen IV (red). Scale bar = 50 μm. (**b**) Microvascular amyloid deposits after digestion and removal of the microvessels stained for fibrillar amyloid using thioflavin S (green). Scale bar = 50 μm. (**c**) Thioflavin T fluorescence showing rapid fibril grow of Aβ42 in the presence and absence of human microvascular amyloid seeds derived from the human brain tissue. (**d**) FTIR spectra of Aβ fibrils formed using soluble wild-type Aβ42 labeled with 1-^{13}C Gly33 added to seeds from human microvascular amyloid deposits show anti-parallel signature.

Supplementary Figure 9

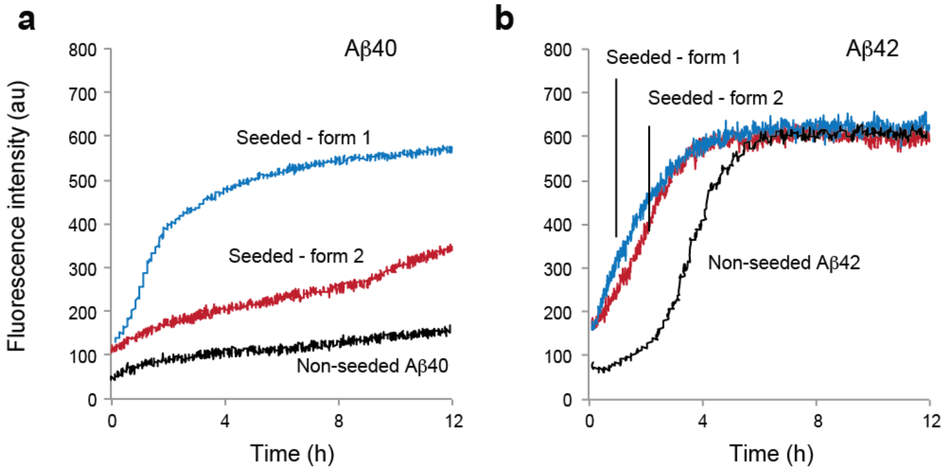

Supplementary Figure 9. Aβ40 and Aβ42 can efficiently cross seed for some fibril structures. Amyloid fibrils have cross-β sheet structure in which the β-strands lie perpendicular to the direction of fibril growth. Within the constraints of this shared general architecture, specific Aβ peptide sequences can generate many different fibril morphologies (as observed by TEM), likely as a result of different molecular structures. Polymorphism in fibril structures can be reduced by multiple sonication – reseeding steps[8]. However, there are several drawbacks to multiple reseeding steps. First, reseeding can potentially lead to an enhancement of fibril structures that are only a minor component of the original mixture. Second, Aβ40 and Aβ42 fibrils often do not cross seed efficiently[8,9]. While Aβ40 is the predominant Aβ isoform in vascular amyloid deposits, there is a significant amount of Aβ42 present. Consequently, re-seeding experiments may discriminate between these two components if there is a difference in the efficiency of fibril growth.

To circumvent these potential issues, the results in the main text are based only from first generation fibrils, i.e. from a single seeding event. Furthermore, we show here that some fibril forms of Aβ42 have the potential to efficiently cross-seed with Aβ40. That is, Aβ42 can form at least two distinct structures[7]. In one structure (form 1), there are only two β-strands that pack in fashion similar to Aβ40[10]. In a second structure (form 2) the C-terminal amino acids bend back to form a third β-strand[11]. Thioflavin T fluorescence measurements were carried out on seeds derived from these two fibril forms using either monomeric Aβ40 (**a**) or Aβ42 (**b**). In the case of Aβ40, seeds derived from fibril

form 1 of Aβ42 can nucleate rapid fibril growth of Aβ40 monomers, but not seeds derived from fibril form 2. The ability of seeds from fibril form 1 to efficiently nucleate Aβ40 growth demonstrates that these two isoforms are not inherently incapable of cross-seeding. Nevertheless, the inability of the seeds of form 2 to efficiently nucleate Aβ40 growth provides a potential explanation for observation that parenchymal plaques are comprised of predominantly Aβ42[11], i.e. the Aβ42 fibril structure in parenchymal plaques may be substantially different from the structure in vascular amyloid. For Aβ42, the seeds from both fibril forms result in rapid fibril growth using Aβ42 monomers. These experiments were undertaken with 40 μM monomer Aβ solutions and 5% seeds.

Supplementary References

1. Tamm, L.K. & Tatulian, S.A. Infrared spectroscopy of proteins and peptides in lipid bilayers. *Q. Rev. Biophys.* **30**, 365-429 (1997).
2. Paul, C., Wang, J.P., Wimley, W.C., Hochstrasser, R.M. & Axelsen, P.H. Vibrational coupling, isotopic editing, and β-sheet structure in a membrane-bound polypeptide. *J. Am. Chem. Soc.* **126**, 5843-5850 (2004).
3. Petty, S.A. & Decatur, S.M. Experimental evidence for the reorganization of β-strands within aggregates of the Aβ(16-22) peptide. *J. Am. Chem. Soc.* **127**, 13488-13489 (2005).
4. Balbach, J.J. et al. Amyloid fibril formation by $A\beta_{16-22}$, a seven-residue fragment of the Alzheimer's β-amyloid peptide, and structural characterization by solid state NMR. *Biochemistry* **39**, 13748-13759 (2000).
5. van der Wel, P.C.A., Lowandowski, J.R. & Griffin, R.G. Structural characterization of GNNQQNY amyloid fibrils by magic angle spinning NMR. *Biochemistry* **49**, 9457-9469 (2010).
6. Qiang, W., Yau, W.-M., Luo, Y., Mattson, M.P. & Tycko, R. Antiparallel β-sheet architecture in Iowa-mutant β-amyloid fibrils. Proc. Natl. Acad. Sci. USA 109, 4443-4448 (2012).
7. Fu, Z., Aucoin, D., Davis, J., Van Nostrand, W.E. & Smith, S.O. Mechanism of nucleated conformational conversion of Aβ42. Biochemistry (2015).
8. Tycko, R. Amyloid Polymorphism: Structural basis and neurobiological relevance. Neuron 86, 632-645 (2015).
9. Portelius, E. et al. Mass spectrometric characterization of brain amyloid beta isoform signatures in familial and sporadic Alzheimer’s disease. Acta Neuropathol. 120, 185-193 (2010).
10. Sato, T. et al. Inhibitors of amyloid toxicity based on β-sheet packing of Aβ40 and Aβ42. Biochemistry 45, 5503-5516 (2006).
11. Xiao, Y. et al. Aβ(1-42) fibril structure illuminates self-recognition and replication of amyloid in Alzheimer's disease. Nat Struct Mol Biol 22, 499-505 (2015).
